# Reproducible parallel inference and simulation of stochastic state space models using odin, dust, and mcstate

**DOI:** 10.12688/wellcomeopenres.16466.1

**Published:** 2020-12-11

**Authors:** Edward S. Knock, Lilith K. Whittles, Pablo N. Perez-Guzman, Sangeeta Bhatia, Fernando Guntoro, Oliver J. Watson, Charles Whittaker, Neil M. Ferguson, Anne Cori, Marc Baguelin, Richard G. FitzJohn, John A. Lees

**Affiliations:** 1 MRC Centre for Global Infectious Disease Analysis; and the Abdul Latif Jameel Institute for Disease and Emergency Analytics (J-IDEA), School of Public Health, Imperial College London, London, W2 1PG, UK; 2 Department of Infectious Disease Epidemiology, London School of Hygiene & Tropical Medicine, London, WC1E 8HT, UK

**Keywords:** Epidemiology, Infectious diseases, Compartmental models, State space model, Particle filter, SMC, MCMC

## Abstract

State space models, including compartmental models, are used to model physical, biological and social phenomena in a broad range of scientific fields. A common way of representing the underlying processes in these models is as a system of stochastic processes which can be simulated forwards in time. Inference of model parameters based on observed time-series data can then be performed using sequential Monte Carlo techniques. However, using these methods for routine inference problems can be made difficult due to various engineering considerations: allowing model design to change in response to new data and ideas, writing model code which is highly performant, and incorporating all of this with up-to-date statistical techniques. Here, we describe a suite of packages in the R programming language designed to streamline the design and deployment of state space models, targeted at infectious disease modellers but suitable for other domains. Users describe their model in a familiar domain-specific language, which is converted into parallelised C++ code. A fast, parallel, reproducible random number generator is then used to run large numbers of model simulations in an efficient manner. We also provide standard inference and prediction routines, though the model simulator can be used directly if these do not meet the user’s needs. These packages provide guarantees on reproducibility and performance, allowing the user to focus on the model itself, rather than the underlying computation. The ability to automatically generate high-performance code that would be tedious and time-consuming to write and verify manually, particularly when adding further structure to compartments, is crucial for infectious disease modellers. Our packages have been critical to the development cycle of our ongoing real-time modelling efforts in the COVID-19 pandemic, and have the potential to do the same for models used in a number of different domains.

## Introduction

To mathematically model a physical or biological process one must develop and test a model, combine it with potentially noisy or poor quality data, and then produce high-quality reproducible results in a computationally efficient manner. This constitutes a multi-disciplinary challenge ^1, 2^. Frameworks which automate common computational and statistical methods can facilitate some of the complex steps in the process, and come with guarantees of efficiency and reproducibility ^3, 4^ – a necessity in modern science, particularly when this science is used in real time to support policy making ^5– 7^. 

When designing these frameworks, it is generally fair to assume that a typical multi-disciplinary modeller is a domain expert and technically minded, but should not have to become a software engineer in order to develop an efficient implementation ^3^. Therefore, as developers of computational frameworks, we should aim to lower the barriers of entry by using a programming language favoured by researchers in the targeted domain, designing a clear and well-documented application-programmer interface (API), and making installation, use and reuse as painless and portable as possible. We can enhance uptake by combining sensible software engineering choices with carefully designed statistical and computational methods. This make design advantages such as speed, unit-tested code and reproducible random number generation as broadly accessible as possible.

With these aims in mind, we describe the development of three libraries in the R programming language ^8^, designed to make the implementation of state space models as easy and reliable as possible. R-like code in the odin domain-specific language (DSL) is automatically transpiled into C++, which is then compiled into a dynamic library with an R interface. The resulting code is portable, and the generated libraries are lightweight and computationally efficient. This procedure offers the performance and careful memory management of compiled code, without requiring the user to have any specialist programming knowledge. Additionally, we include an R package, mcstate, which provides routines for common inference and prediction tasks. Compiled models also link with functions directly callable from R, so users are free to develop more flexible uses and inference procedures using R programming. We provided detailed examples of applying these tools to simple stochastic epidemic models, both here and in the package documentation.

## Methods

### Implementation

State space models relate input, output and state over time using a probabilistic model linking states at subsequent time steps, and can be used to model a broad range of processes. Below we describe how four major components of our framework, bundled as packages for the R programming language, can be used to implement these models. All of the packages require 100% code coverage of tests, and include unit tests designed by software engineers and integration testing (of entire analysis pipelines) designed by statisticians and epidemiologists. Code to reproduce this analysis and all of the plots in this paper can be found at https://github.com/mrc-ide/odin-dust-plots.

### odin – A DSL for R programmers to write efficient state space models

Packages which allow users to implement their own model code straddle a difficult dichotomy: making models fast and efficient to use, and making models fast and efficient to develop. Using an intermediary DSL is one approach to this issue, also applied in the popular JAGS and stan packages, as this offers a bridge between the more familiar language style used to develop models, and the compiled languages preferred for running large models ^9^. Compared to writing directly in a compiled language, DSLs have the further advantage that we can design error messages which are domain specific, rather than the sometimes convoluted errors from compilers (which are necessary due to their generality). 

odin is a DSL we have been developing since 2016, and has been used to model both continuous and discrete time models. odin is syntactically similar to R, and by taking advantage of R’s ‘non-standard evaluation’ ^10^ presents users with a simple interface for describing sets of equations. The general approach of the package is that the user expresses their problems as a set of mathematical relationships, modelled as assignments to form a directed acyclic graph (DAG). odin then sorts that graph and transpiles the equations to code in a chosen target language. Users can therefore write their equations in any order, which is more similar to mathematical formalism than declarative programming. 

For use with ordinary differential equations (ODEs), odin transpiles to C code ( https://mrc-ide.github.io/odin/) or JavaScript ( https://mrc-ide.github.io/odin.js). For the models in this paper, we focus on transpilation of discrete time stochastic models into C++ using a framework (dust) that we describe below. In both cases, odin has an R interface, allowing its standalone use, or inclusion in R packages.

By eliminating logic and ‘general programming’ (such as defining types, writing loops), the models become relatively simple sets of mathematical truths that map closely to the scientific domain, yet remain efficient to solve. In addition to scalar relationships, odin provides a syntax designed to easily add structure to compartments - for example to represent age or transmission classes and the interaction between these without requiring explicitly written loops. Arrays representing structure classes are written implicitly with indices, meaning models can easily be extended. An example of adding age structure is shown in the Use Cases. 

Specific functions available to the user beyond basic arithmetic include the random number generation functions detailed below, and optimised sums over state items. We include most of the functions available in the Rmath library ^8^ in odin, which are documented at https://mrc-ide.github.io/odin/articles/functions.html. A subset of these functions are currently available for dust, and we intend to continue to expand this support.

### mcstate – An R package implementing common SMC inference techniques, using odin models

The odin DSL gives modellers the ability to write a fast state space model in R, and interact with it in a number of fundamental ways. While some users may wish to implement their own inference techniques using these building blocks, we expect that most will use the standard methods we provide and test in the mcstate R package, as mcstate provides all the necessary routines for statistical inference from these models.

The key additional programmatic elements that mcstate provides for state space modelling are the definition of some observed data, and an observation function, which defines the log-likelihood of the observed data given the underlying model state. As the observation function is written directly in R, the user is free to define this however they choose, as long as it accepts the model state and some data as arguments. This may therefore use the state history stored in R, for example to compute the change in sizes of compartments and take advantage of large library of built-in functions. Some typical observation functions are described in the Use Cases below.

The mcstate package provides a particle filter implementation, also known as SMC (Sequential Monte Carlo) ^11, 12^, which enables efficient parameter inference with high-variance model runs. A dust model is run forwards in time for a number of particles ( *n*). At each step where observations are available these are compared to the data, and a likelihood weight computed for each particle. The *n* particles at observation time step *j* are then resampled with replacement, with probabilities corresponding to their likelihood weight, to select *n* particles to be run to observation time step *j*+1 ^13^. This SMC process runs the update function for all particles, resamples, and continues until the final observation is reached. This final state is sometimes referred to as a ‘nowcast’. A function is provided to convert observational data into the correct format for the particle filter. 

With this technique for combining potentially stochastic observations with a stochastic model, a marginal likelihood given model parameters can be produced. We can use the log-likelihood derived from the particle filter to perform Bayesian inference on model parameters. To do this, mcstate uses Markov Chain Monte Carlo (MCMC) over runs of the particles filter, known as pMCMC ^14, 15^. Currently mcstate supports standard Metropolis-Hastings MCMC: *m _c_* independent chains are run, with care taken to ensure independent random number streams. The user provides prior distributions as functions in R, and a variance-covariance matrix for the proposals. Proposals at each step are drawn from a multivariate normal distribution, are reflected if outside of a specified minimum or maximum, and discretised if required. 

We also support forecasting from the final observation position. The estimate of the posterior distribution produced by an MCMC run is sampled from by sampling particles with replacement. These runs extend the original model run by applying to state update functions until the time has reached the required length, picking up from the final random number generator (RNG) state of the particle. If further forecasts are required, a new RNG state is seeded from R’s internal state, to avoid producing identical forecasts from the same streams of psuedorandom numbers.

### dust – A C++ template library for driving parallel stochastic models from R

**Figure 1.**
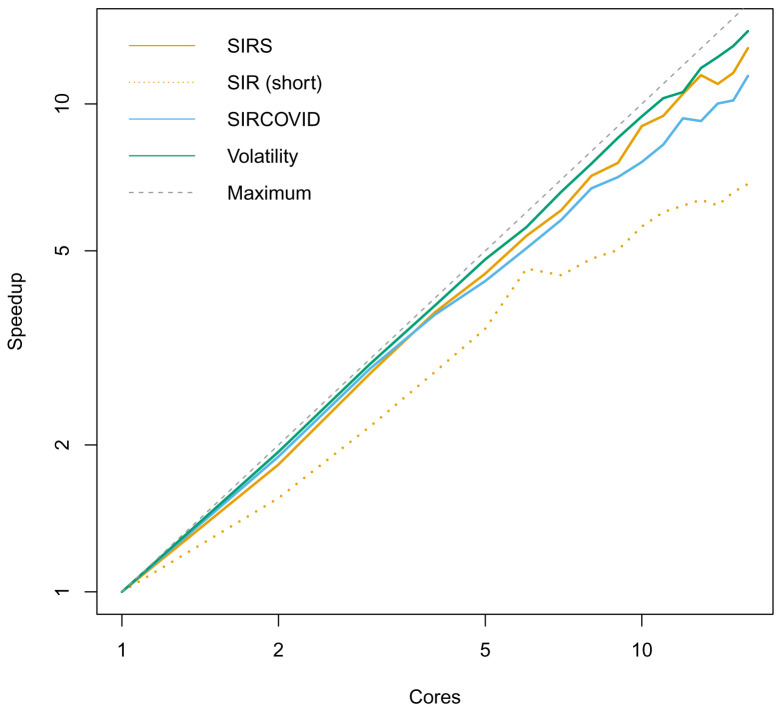
Speedup of dust simulations as number of threads increases, on a log-log scale. Models are described in the Use Cases. Speedup is defined as the ratio of wall time (total program time) taken to the wall time using a single thread. The models were run for 5 × 10 ^5 ^ steps using *n _P_* = 10 ^3 ^ to artificially increase the amount of computation. The SIRS model has an additional R to S transition to make the infection endemic, otherwise the infection dies out, and no significant processing is used. The ‘SIR (short)’ model demonstrates a fall in performance for short running models, in this case with 10 ^3^ steps and *n _P_* = 10 ^2 ^. We also ran the SIRCOVID model inference with *n _P_* = 10 ^2 ^ for 10 ^3^ MCMC steps. The consistent speedup demonstrates that the multicore use is effective, even when running a full pipeline with a particle filter and evaluation of a log-likelihood in R.

The above statistical techniques are computationally demanding, particularly as models become more complex, and to run them in a reasonable time-frame we needed an efficient engine to run stochastic models. Noting that between each observation step every particle is independent, we designed a system that could simulate the particles in parallel, so that we could take advantage of the increasing availability of multi-core CPUs. Our solution is implemented in the R package dust. To inferface with dust, users must provide an update function, which is the core of the model, and we provide some examples below. While the user can write this in C++, we expect most users to use odin.dust to generate this code automatically. 

dust uses two main abstractions to represent and run state space models: Particle and Dust. A Particle object is a single trajectory simulated from the model and a Dust object is a collection of *n _P_* Particles with the same model parameters and initial conditions, but different trajectories due to the inherent stochasticity of the system being simulated. Internally, Particles within a Dust object can be shuffled and sampled, to support particle filtering methods. A Dust object can be run forward in discrete steps, moving all Particles forward the same number of steps. This is the main computation in dust, and is parallelised on up to *n _P_* CPU cores using OpenMP ^16^. Using the static schedule to evenly distribute particles across cores gave close to linear increase in performance with the number of cores with long running models described in the Use Cases ( Figure 1).

In addition, we tested the speedup of a large SEIR (susceptible-exposed-infected-recovered) model for COVID-19 transmission in the UK, implemented using the odin DSL, using dust and mcstate to infer its parameters. Due to 19 age-classes which add structure to most of the model compartments, the model has around 1000 compartments in total. The computation time of running simulations of this model is therefore dominated by many random number draws, and so can be efficiently parallelised using the techniques described above. We confirmed this using a CPU profiler, finding that at least 61% of processing time being spent in the rbinom() function (described below). This dust model and its interface is named sircovid, and its code and documentation can be found at https://mrc-ide.github.io/sircovid. Running with *n _P_* = 100 for 2000 steps, one MCMC chain on a laptop took around 1 hour with a single core, and showed roughly linear speedup with number of cores when used for either extra particles or extra chains. 

On a personal computer, users can employ up to *p* cores to run the *n _P_* particles of dust objects, or use these cores to run the *m _c_* chains of mcstate, as long as *n _P_m _c_ <*= *p*. On distributed infrastructure with disconnected nodes each with many cores, memory is shared on a node, but not between nodes. In this case, the optimal use of resources is to run *n _P_* particles on a single node, using all *p* of its cores. The *m _c_* chains, or parameter sets, can be independently run on up to *m _c_* separate nodes, and their results combined using the provided functions. This is detailed in the ‘parallelisation’ vignette in the mcstate package.

The C++ source of the package exists largely as a header-only template library. This has the advantage that no platform-specific library code is needed for the generation of models, simplifying the installation process and giving wider support across operating systems and hardware architectures. Furthermore, compiling and optimising is always done using the whole model code in a single unit. The compilation is launched from within R, and once finished gives a shared object with R methods to run, shuffle and extract state from the underlying particles.

If writing a model directly in C++ there are some minimal interface requirements, which constrain the types of model which can be run through dust. Specifically, the user must provide a model class to dust with the following functions:

- initial() – Loads data passed from R to set the initial state and model parameters.
- size() – Computes the size of the model state for a single particle (number of compartments).
- update() – Updates the model for a single timestep. This may only depend on the previous state i.e. it must be Markovian. The function has access to the model parameters, timestep and random number generator functions.

More flexible simulation runs than provided by mcstate, for example from running counterfactuals, are straightforwardly supported by direct use of the dust object in R, while providing alternative parameter sets.

### Random number generation

Generating random numbers from common distributions is a cornerstone of designing the model update function for many epidemiological models. This is also true computationally – when profiling a complex compartmental model for COVID-19 transmission, we found that at least 61% of program time was spent computing random deviates. R’s default number generator is not able to operate in parallel, which meant that using the standard library functions for generating random deviates from common distributions was not an option. We therefore took particular care with the design of a parallel random number generator used by dust. 

Random number generation on a computer produces a stream of pseudorandom numbers, usually integers in a specified interval, which are uncorrelated, but deterministic given a set starting point (the ‘seed’). For stochastic model simulations there are two main considerations: running independent model realisations, and making results reproducible. Using the same seed for different particles will give identical results, and will break the assumptions of downstream inference methods ^17^. However, using the same seed for an entire set of particles is desirable, so results can be reproduced. We also wish for our RNG implementation to ‘play-fair’, which means that results are independent of the specific hardware used, and the degree of parallelisation ^18^. This is needed for scientific reproduciblity and effective debugging – knowing a change in results is attributable to a change in model, and not a change in randomness is vital for developing models. 

The simplest solution, which is the default in R, is to run particles serially with each subsequent particle continuing from a single random number stream. However, serial particles places a limit on parallelisability. A frequent way around this is to make *m* RNGs for each parallel thread, and seed each one with a new but pre-specified seed. However, as the state space of the RNGs is highly non-linear and chaotic, it isn’t possible to predict at which point a given seed will enter the stream, shared between all RNGs with the same design. For even modest simulation lengths and levels of parallelism, this can lead to correlated streams of random numbers, again breaking downstream inference assumptions. 

**Figure 2.**
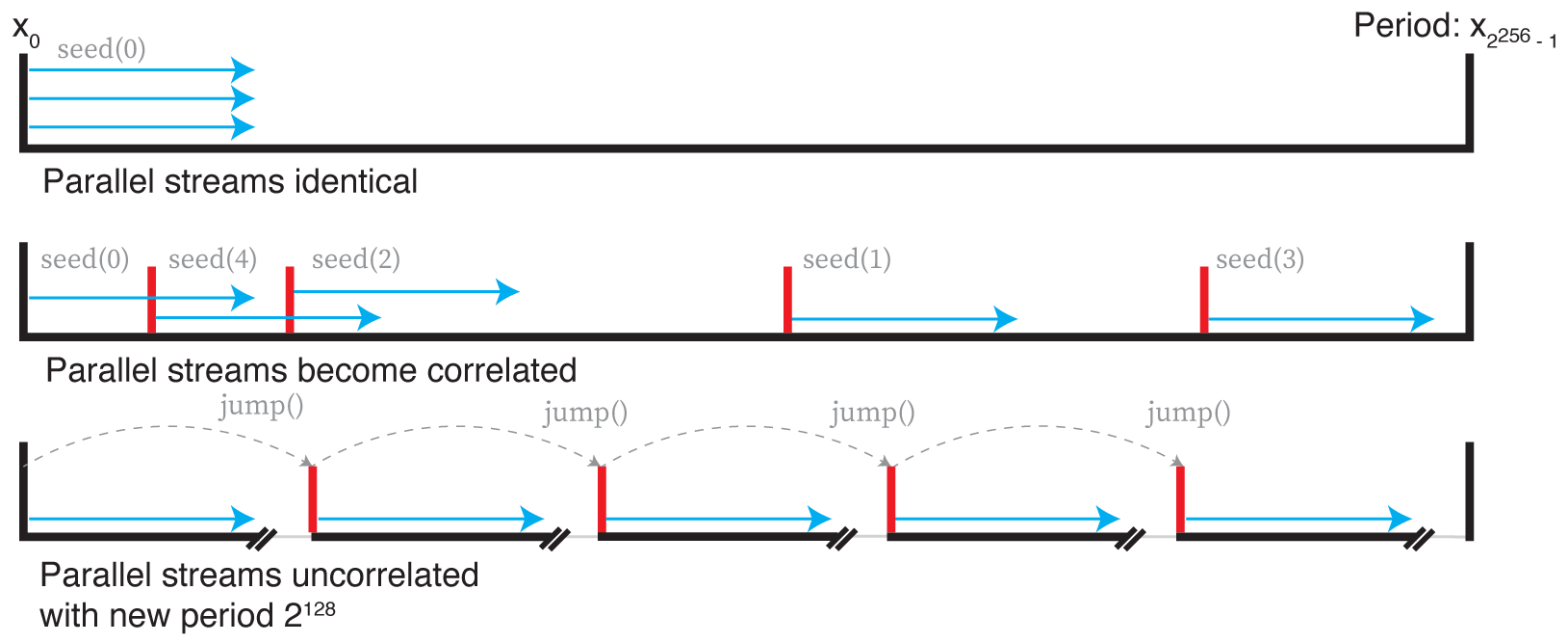
Top: parallel random number streams with the same seed are identical. Middle: parallel random number streams with different streams can quickly become correlated. Bottom: using the jump() function of the Xoshiro generator moves forward 2 ^128^ steps, giving evenly spaced, uncorrelated streams of random numbers.

One solution is to create *m* RNGs which can be advanced or ‘jumped’ a set number of steps. If each thread’s RNG consumes *k* random numbers, then advancing each RNG *m _i_* by *ik* steps before running will ensure independent streams for each process. If *k* is not known, this becomes more difficult. The approach we follow here uses a new class of RNGs known as Xoshiro (XOR, shift, rotate) ^19^. These generate a stream of pseudorandom integers in the interval [0,2 ^64^) with a period 2 ^256^. Generation is very fast, but importantly also implements a jump() function which advances the generator by 2 ^128 ^steps in a time comparable to a single random number draw. Applying this allows the initialisation of up to 2 ^128 ^ RNGs, each capable of drawing a stream of up to 2 ^128 ^pseudorandom numbers before correlating. These concepts are summarised in Figure 2.

Random number streams should not vary based on the number of threads used in a specific run, and should instead always be reproducible from the same single seed. Therefore every particle *p* has its own RNG, rather than every thread; this is feasible given the relatively small state (256 bits) used by Xoshiro compared with generators such as Mersenne Twister (2560 bits) ^20^. A single 64-bit integer seed is passed from R, with the remaining three chunks of 64-bits-of-state set pseudo-randomly from this using the splitmix64 algorithm ^21^. These 256 bits are used to set the initial state of a xoshiro256** generator. The RNG state for particle *i* begins with this state, after applying the jump() function *i* times. 

Many off-the-shelf parallel random number generators are aimed at repeated generation from a distribution with the same parameters, including those in the C++ standard library. This is ill-suited to state space models, where distribution parameters typically change between every generation, and between every particle. The TensorFlow ^22^ code base contains suitable implementations, but would be a large and complex dependency, difficult to make fully compatible with R. Therefore, we added code which uses the Xoshiro generator to transform into random deviates from statistical distributions:

- runif( *a*, *b*) - A uniformly distributed real number in the interval [ *a*, *b*), by dividing RNG state by its maximum value of 2 ^64^.
- rnorm( *µ*, *σ*) - A normally distributed real number with mean *µ* and variance *σ* ^2^, by applying the Box-Muller transform to runif(0, 1) ^23^.
- rbinom( *n*, *p*) - A binomially distributed integer given *n* trials and a probability of success *p*. Uses inversion transform sampling with exponentiation by squaring if *np* < 10 ^24^, or transformed rejection sampling with the ‘BTRS’ algorithm otherwise ^25^.
- rpois( *λ*) - A Poisson distributed integer given rate *λ*. Uses Knuth’s algorithm for *λ < *10 ^26^, and transformed rejection sampling otherwise ^27^.

All of these methods are optimised for when the parameters of the distribution change every sample, as expected with stochastic state space models. We plan to add other random number distributions as required, though these were sufficient for all models currently tested. This is all implemented using C++ to be bundled with dust, and can directly be accessed from R.

### Operation

We provide an overview of the typical workflow runs through these packages in sequence. Steps 1 and 2 are model simulation, 3 to 7 for model inference, and 8 for forecasting. In cases where models are not being fitted to data, a dust generator can be used directly to simulate from the model with given parameters, and this process ends after the second step. If any model parameters are being inferred from the data steps 1-7 are required, with step 8 an optional addition if a further forecast using the inferred values is needed. Users with more complex needs not met by an odin model may also skip step 1, and write a dust target in C++ directly, while still using the dust RNG library and functions if required.

1. *Write a model in the odin DSL*. Markov models will define a set of update() functions which together give the state at *t* +1 by operating on the state at *t*.
2. *Compile the model into a dust object*. Using odin.dust, this will create a shared library and R interface. This will turn the odin code into a single update() update function usable by dust. This step may be performed iteratively within an interactive session for rapid development, or by creating an R package for more robust development.
3. *Write an observation function*. This will compare model simulations to data, and calculate the log-likelihood of the model run given the data. Users are free to implement this however they wish, and can leverage any R function or package to do so.
4. *Load observed data*. Typically this will be time series data in R, as a data.frame. Use the included functions in mcstate to convert this into input for the particle filter, potentially adding an offset and intermediate steps without observations.
5. *Create a particle filter object*. This uses the dust generator, observation function and observed data.
6. *Set parameters*. Define prior functions and pMCMC jump size for unknown parameters; set the values of known parameters.
7. *Run a pMCMC*. Using parameters and the particle filter, this will return posterior density estimates for each unknown parameter.
8. *Forecast trajectories*. If a forecast is required, run the predict() function in mcstate after the pMCMC, which will create simulated trajectories for each particles past the end of the data, while sampling parameters from the posterior.

System requirements for running are:

- R (>=v3.5.0)
- A C++ compiler such as gcc or clang is needed to compile dust models.
- An appropriate OpenMP library (>=v3.0), including a C++ compiler supporting the -fopenmp option, such as gcc (>=v4.0) or clang (>=v3.9). If OpenMP is not available, models will still compile, but parallelisation of particles will not be supported.
- odin, odin.dust, dust and mcstate, all of which can be installed with standard tools. Here, we used odin v1.0.8; odin.dust v0.1.1; dust v0.5.3; mcstate v0.2.15.

All our R packages are available for R on Linux, OS X and Windows, and are open source using the MIT licence.

## Use cases

Here we exhibit some brief examples of state space models, followed by a typical use case in epidemiology. We demonstrate the ease of use of these packages as this model becomes more complex, and is expanded to more realistic scenarios. These examples are included with the dust package, and detailed vignettes to reproduce the analysis here are included with the mcstate package.

### Basic stochastic models

A basic model for volatility, which is a broadly used concept in finance describing randomly distributed variance of an asset’s price *x*, is given by:

$$dx_{t}=axdt+\sigma dW_{t}$$

where *α* is the constant drift, *σ* is a constant volatility and *dW _t_* is a Weiner process with zero mean and a variance of one ^28, 29^. Given the properties of Weiner processes (Brownian motion), the update function using the Euler-Maruyama method is:

$$\begin{array}{c} x_{t+1}=ax_{t}+\sigma *w\\ w\sim N(0,1) \end{array}$$

which in the odin DSL is simply:


update(x) <- x * alpha + sigma * rnorm(0, 1)
initial(x) <- x0
alpha <- user(0.91)
sigma <- user(1)
x0 <- user(0)


**Figure 3.**
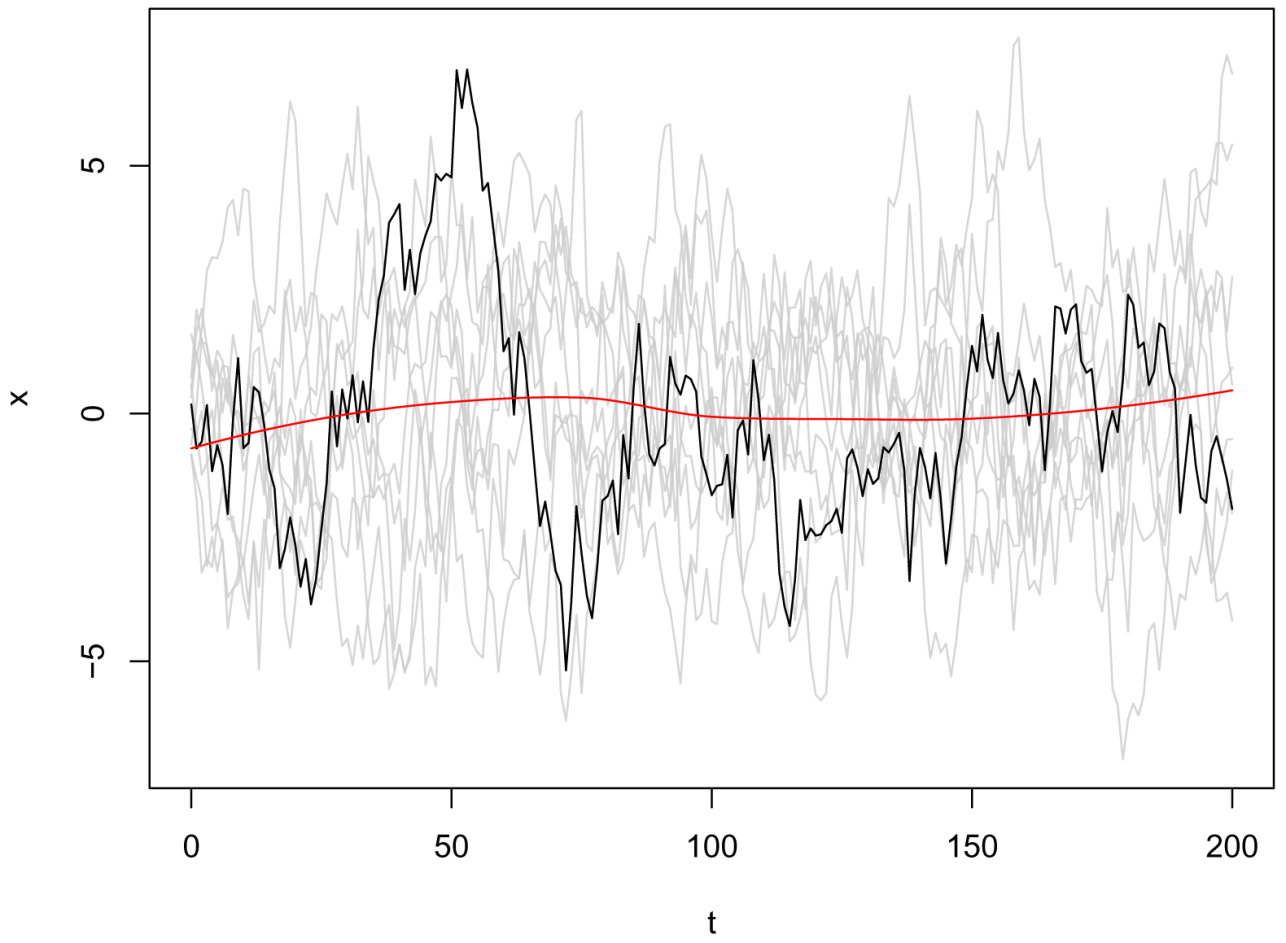
Plot of the *x* state from ten independent realisations (particles) from the volatility model, with an example trajectory highlighted in black, and the smoothed mean value from all particles in red.

The user() syntax specifies this will be a parameter provided through the R interface, either directly or through an inference method (such as mcstate). Default values can be set in brackets. Multiple realisations of this model can be run through dust ( Figure 3) as follows:


gen <- dust::dust_example("volatility")
# more generally:
# gen <- odin.dust::odin_dust("volatility.txt")

vol <- gen$new(data = list(alpha = 0.91),
               step = 0,
               n_particles = 10,
               n_threads = 4L,
               seed = 1L)
vol$run(10)


This is included as an example in the dust package, but users can load their own model code by calling odin_dust on the text file containing their model. This example illustrates running ten particles, ten timesteps forwards parallelised over four threads. The seed provided gives reproducible, uncorrelated runs for each of the particles.

### Stochastic SIR model

Models of infectious disease transmission – such as the susceptible-infected-recovered (SIR) model ^30, 31^ – are an obvious use of our packages. Typically these models can be expressed in three related forms: ODEs, stochastic differential equations (SDEs) or as a continuous time Markov chain (CTMC) ^32^. Their solutions have different properties: ODEs are fast to solve numerically, and are deterministic given a set of initial conditions; SDEs give stochastic solutions each time they are solved which better represent the variance in real-world systems, and still have efficient numerical solvers; CTMCs best represent the discrete nature in small populations and correctly model absorbing states, but are computationally more intensive to realise trajectories from ^33^. For the remainder of this section we focus on stochastic models, particularly CTMC formulations of infectious disease transmission models. A simple definition (using the ODE formalism) of the SIR model is:

$$\begin{array}{l} \begin{array}{c} \frac{dS}{dt}=-\beta \frac{SI}{N} \end{array}\\ \begin{array}{c} \frac{dI}{dt}=\beta \frac{SI}{N} \end{array}-\gamma I\\ \frac{dR}{dt}=\gamma I \end{array}$$

*S* is the number of susceptibles, *I* is the number of infected and *R* is the number recovered; the total population size *N* = *S* + *I* + *R* is constant. *β* is the infection rate, *γ* is the recovery rate.

This model can be discretised in time steps of width *d t* using the following update equations for each time step ^32– 34^:

$$\begin{array}{l} S_{t+1}=S_{t}-n_{SI,t}\\ \;I_{t+1}=I_{t}+n_{SI,t}-n_{IR,t}\\ R_{t+1}=R_{t}+n_{IR,t} \end{array}$$

where the number of individuals moving between compartments are given by drawing from binomial distributions:

$$\begin{array}{l} n_{SI,t}\sim B(S_{t},\;1-\exp(-\beta \frac{I_{t}}{N}\cdot dt))\\ n_{IR,t}\sim B(I_{t},\;1-\exp(-\gamma \cdot dt)) \end{array}$$

the binomial distribution is used as there are *n* trials, one for each individual in the compartments, who move with per-capita transition probability *p*. In a single time step, *p* can be calculated as 1 − *e* ^*λ*· *dt*^ where *λ* is the transition rate, as in a Poisson process time between events is exponentially distributed.

These equations can be written in the odin DSL as:


## Definition of the time-step and output as "time"
dt <- user(1)
initial(time) <- 0
update(time) <- (step + 1) * dt

## Model parameters (default in parenthesis)
beta <- user(0.2)
gamma <- user(0.1)

## Initial conditions
initial(S) <- 1000
initial(I) <- 1
initial(R) <- 0

## Core equations for transitions between compartments:
update(S) <- S - n_SI
update(I) <- I + n_SI - n_IR
update(R) <- R + n_IR

## Individual probabilities of transition:
N <- S + I + R # total population size
p_SI <- 1 - exp(-beta * I / N * dt) # S to I
p_IR <- 1 - exp(-gamma * dt) # I to R

## Draws from binomial distributions for numbers changing between
## compartments:
n_IR <- rbinom(I, p_IR)
n_SI <- rbinom(S, p_SI)


This would be saved as sir.R and then compiled with odin.dust::odin_dust("sir.R").

Initial conditions here are fixed, but they can also be added to the user() group to be set from R for each run. For infectious disease models, users may follow the guidance in the odin documentation on discretising ODE models using appropriate random number draws ( https://mrc-ide.github.io/odin/articles/discrete.html). 

**Figure 4.**
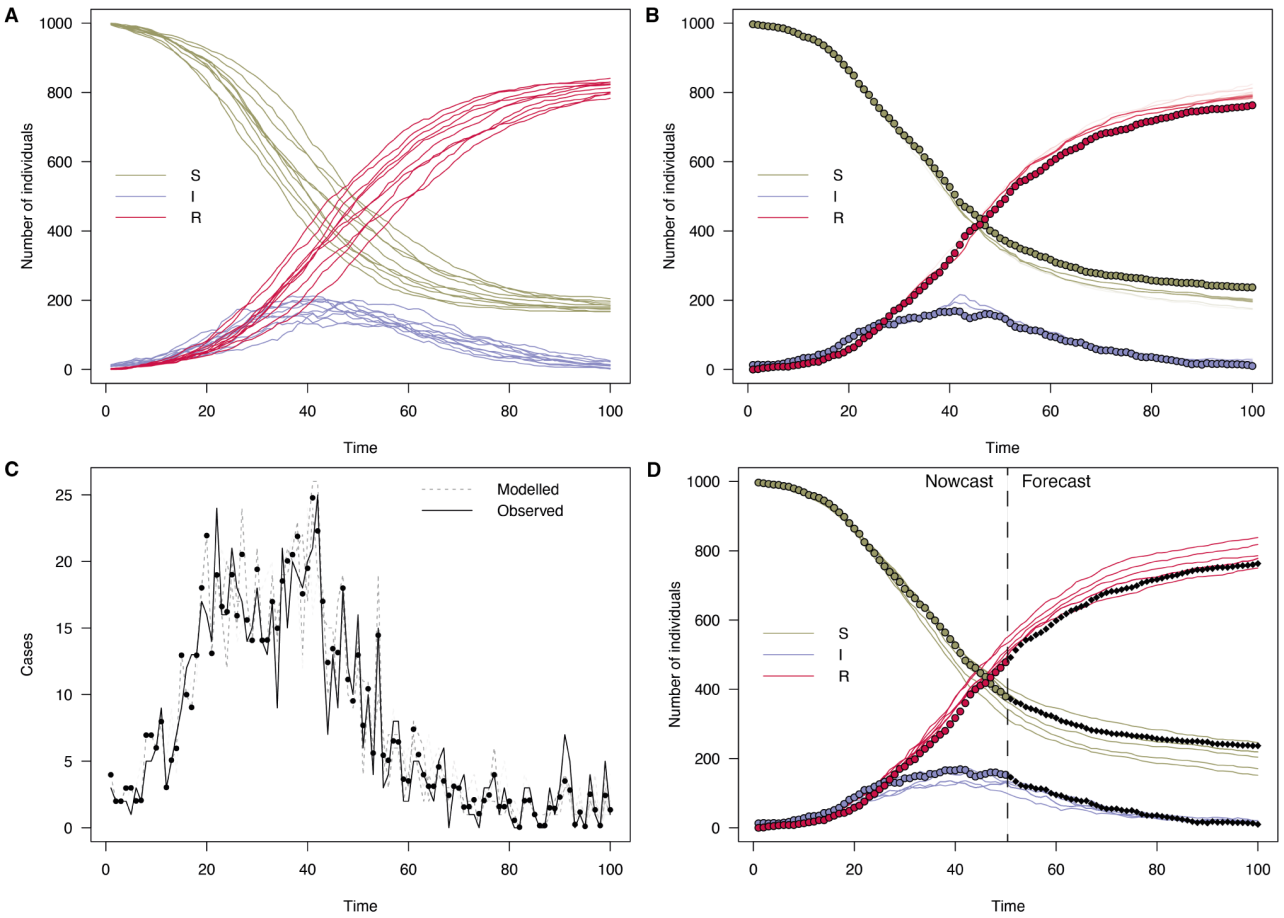
Plots of the number of individuals in the *S*, *I* and *R* compartments over time in an SIR model with *β* = 0.2 and *γ* = 0.1. **A**: 10 particles run forward for 100 time steps. **B**: Solid points are data generated from the model from which simulated case counts were produced. Lines are 100 particles run forward using a particle filter with this data. **C**: Daily case incidence data which was fitted to (black), and modelled incidence of 100 particles (grey). Average of particles shown as points **D**: Extending the particle trajectories forward in time by simulating the model forward with parameters sampled from the estimated posterior. Here, only the first half of the time series was used to show a more uncertain part of the epidemic, subsequent real points are shown in black.

This model can simulate an epidemic forward in time using fixed parameters, as shown in Figure 4. We can also use this model to demonstrate the inference process (steps 3-8 in Operation), showing how the transmission rate *β* and recovery rate *γ* (as well as $R_{0}=\frac{\beta }{\gamma }$ ) can be inferred from observed daily case counts *y _t_*. For each day *t*, the number of expected new cases (if all cases are observed) can be assumed to be Poisson distributed with mean *y _t_* , so an observation function can be written by taking the log of a Poisson probability mass function at *k _t_* = *S _t_*_−1_ − *S _t_* = *n _SI,t_*. This can easily be achieved using the builtin R function dpois() (one of many probability-distribution functions available to users):


case_compare <- function(state, prev_state, observed, pars = NULL) {
  cases_modelled <- prev_state[1, , ] - state[1, , ]
  dpois(incidence_observed, observed$cases, log = TRUE)
}


In the state array, the first dimensions is over model compartments, the second dimension is over particles, and the third over time. So the index ‘1’ extracts the first state, the number of susceptibles. Multiple data-streams can straightforwardly be added by noting that log-likelihoods sum, as long as they are conditionally independent. That is, given the simulated quantities in the model, the observed quantities are assumed to be independent, and have independent noise. So a similar log-likelihood component could be defined based on deaths, in a model with these compartments, and added to the existing function. If a data stream isn’t measured at a particular time point, it simply contributes zero to the log-likelihood.

A particle filter can then be set up using mcstate (steps 4 and 5), choosing the number of particles, and formatting observational data appropriately with the built-in function particle_filter_data(). The observations must be evenly spaced, though missing observations are permitted. The step size in the data is defined to be one, and $\frac{1}{dt}$ update steps are taken between each observation.


n_particles <- 100
dt <- 0.25
data <- particle_filter_data(data, time = "day", rate = 1/dt)
filter <- particle_filter$new(data = data,
                              model = sir,
                              n_particles = n_particles,
                              compare = case_compare)


The right panel of Figure 4 demonstrates using this observation function with case data simulated from the model. Only those trajectories consistent with the data are continued forward at each step, and a final log-likelihood of the model parameters given the data is produced. 

Steps 6 and 7, which are used to estimate the posterior density for *β* and *γ*, are achieved by defining sampling distributions for the parameters, and running a set of MCMC chains. Priors for each parameter can be added at this stage, shown on *γ* for demonstrative purposes:


beta <- pmcmc_parameter("beta", 0.2, min = 0)
gamma <- pmcmc_parameter("gamma", 0.1, min = 0,
                         prior = function(p)
                           dgamma(p, shape = 1, scale = 0.2, log = TRUE)
                        )
proposal_matrix <- diag(2) * 0.01^2
mcmc_pars <- pmcmc_parameters$new(list(beta = beta, gamma = gamma),
                                  proposal_matrix)
                            
pmcmc_run <-
  pmcmc(
    mcmc_pars,
    filter,
    n_steps = 2000,
    save_state = TRUE,
    save_trajectories = TRUE,
    progress = TRUE,
    n_chains = 4
  )


Noting that in the SIR model $R_{0}=\frac{\beta }{\gamma }$ , we could alternatively directly sample *R*_0_ instead of *β*. This is achieved by also supplying a transformation function, which takes parameters being sampled in the MCMC, and returns a list of user input parameters needed by the odin model:


R0 <- pmcmc_parameter("R0", 2, min = 0)
parameter_transform <- function(pars) {
    beta <- pars[["gamma"]] * pars[["R0"]]
    gamma <- pars[["gamma"]]
    list(beta = beta, gamma = gamma)
}


**Figure 5.**
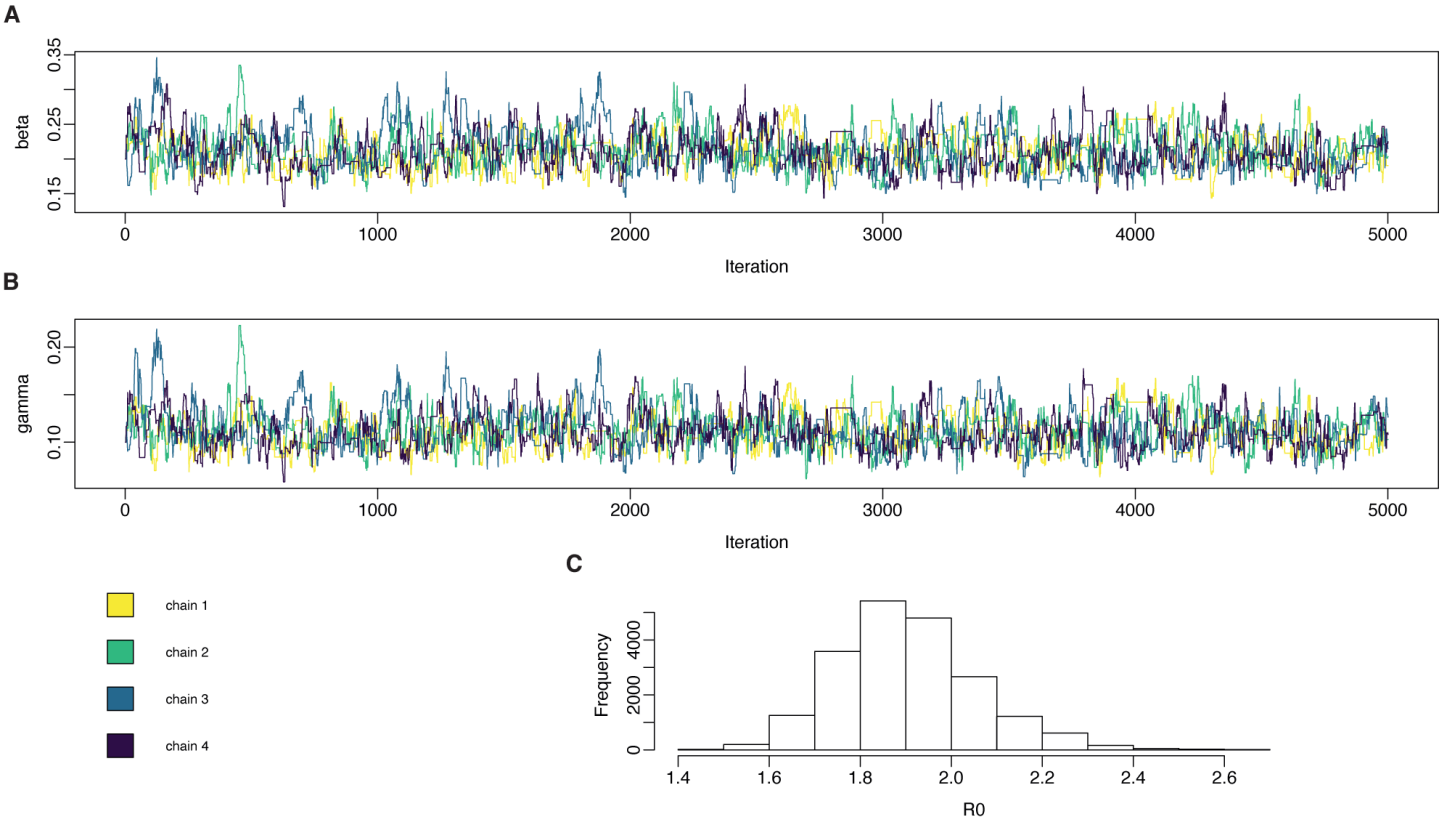
Inferring parameters in compartmental models: the SIR model fitted to simulated daily case data. The particle filter was set up as specified, and four independent chains were run, each chain taking 2 × 10 ^3^ samples for the SIR model. In the SIR model, true values are *β* = 0.2, *γ* = 0.1, *R*_0_ = 2. **A**: posterior samples from *β*. **B**: posterior samples from *γ*. **C**: marginal posterior distribution for $R_{0}=\frac{\beta }{r}$ .

Posterior distributions for *β* and *γ* are available from the pmcmc object, and are plotted for this example in Figure 5. They can be loaded directly into standard MCMC analysis packages such as coda ^35^ to produce diagnostics such as effective sample size, the Gelman-Rubin diagnostic $\hat{R}$ . As is typical with MH sampling, the proposal distribution will likely need to be tuned to get an appropriate acceptance rate, typically thought to be 0.234 for high dimensional problems ^36^. One automated way to do this is to run the chains for a short time, calculate the variance-covariance matrix among the samples, and use this as the proposal kernel.

Finally, producing a forecast past the end of the data (step 8) is achieved simply by calling predict() on the above MCMC object (as shown in the final panel of Figure 4).

### Adding age-structure to the SIR model

This model can be extended to add more flexibility or more specificity when modelling the biology of different diseases. For example: adding new compartments to represent other disease states; compartments which represent spatial or age structuring of the population; or delay distributions which model different rates at which individuals pass through disease pathways. By matching the compartments and the transitions between them to the disease being studied, infectious disease epidemiologists can flexibly and accurately model a wide variety of real world processes.

 As an example of how the basic code given above can be extended, we demonstrate how the SIR model can incorporate age-structure into each of its three compartments. Adding age structure to the model consists of the following steps, which turn variables into arrays:

- Define the number of age categories as a user parameter *N*_age_.
- Add age structure to each compartment, by adding square brackets to the left hand side of each declaration.
- Modify the right hand side of each declaration to use quantities from the appropriate compartment, by adding indices *i* and *j* as needed. These will automatically be turned into loops by odin.dust.
- Where an age compartment needs to be reduced into a single compartment/variable, we use sum (though further array reduction functions are available).
- Define the dimensions of all arrays, for example by setting dim(S) <- N_age.

Alone, this would simply give *N _age_* independent processes equivalent to the first model, scaled by the size of the population in each age category. To actually make this useful, some form of interaction or transitions need to be added between the compartments. An example of this would be to add an age-specific contact matrix *m*, which defines a different force of infection *λ* for each age group. This is calculated by

$$\lambda_{i}=\frac{\beta }{N}\cdot \sum_{j\text{=1}}^{N_{\text{age}}}I_{j}m_{ij}\;(1)$$

In the odin DSL:


m[, ] <- user() # age-structured contact matrix
s_ij[, ] <- m[i, j] * I[j]
lambda[] <- beta / N * sum(s_ij[i, ])


The probability of infection of a susceptible is then indexed by this force of infection:


p_SI[] <- 1 - exp(-lambda[i] * dt)


Putting this all together, the key components of the age structured SIR model is as follows (omitting initial conditions and parameter values from the unstructured SIR model for simplicity):


## Core equations for transitions between compartments:
update(S[]) <- S[i] - n_SI[i]
update(I[]) <- I[i] + n_SI[i] - n_IR[i]
update(R[]) <- R[i] + n_IR[i]

## Individual probabilities of transition:
p_SI[] <- 1 - exp(-lambda[i] * dt) # S to I
p_IR <- 1 - exp(-gamma * dt) # I to R

## Force of infection
m[, ] <- user() # age-structured contact matrix
s_ij[, ] <- m[i, j] * I[i]
lambda[] <- beta / N * sum(s_ij[i, ])

## Draws from binomial distributions for numbers changing between
## compartments:
n_SI[] <- rbinom(S[i], p_SI[i])
n_IR[] <- rbinom(I[i], p_IR)

## Total population size
N <- sum(S) + sum(I) + sum(R)

# Array dimensions
dim(S) <- N_age
dim(I) <- N_age
dim(R) <- N_age
dim(n_SI) <- N_age
dim(n_IR) <- N_age
dim(lambda) <- N_age
dim(m) <- c(N_age, N_age)
dim(s_ij) <- c(N_age, N_age)


The contact matrix *m* is input from R. One choice is to base it on the POLYMOD survey, which can conveniently be loaded in through the socialmixr R package ^37^. This can be sent as input to the model by adding it to the list returned by a transformation function. While we use 1- and 2- dimensional structures here, odin currently supports up to 8 dimensions, allowing for concise description of structured models (at the time of writing, we know of use of up to 4 dimensions).

### Comparison with alternative packages

Although state space models can in some cases be analysed using a general Bayesian hierarchical framework such as JAGS ^38^ or stan ^39^, care needs to be taken with state space models as trajectories can rapidly diverge from time-series data with stochastic update functions, and fitting may become slow ^40^. Two previous packages which aim to solve these issues in a similar way are pomp (partially-observed Markov processes) ^41^ and libBi (library for Bayesian inference) ^42^. 

Both packages use similar concepts to dust and mcstate, requiring users to define a Markovian update function ( rprocess() in pomp; transition() in libBi), an initial state ( rinit() in pomp; initial() in libBi) and a observation function ( dmeasure() in pomp; observation() in libBi). These packages both support simulation and inference from the model using the same overall methods as mcstate, and additionally support some optimisation procedures such as maximum likelihood or particle perturbation.

These packages also support some link between interpreted and compiled languages. In pomp, the interface is in R, but it also any of these functions may be written either in R or in C. This allows us to demonstrate the advantage of using compiled functions to simulate from models on an even footing. Using an SIR model coded in pomp using R functions, and and equivalent implementation in pomp coded using C function, a speedup of around 100x was seen comparing C to pure R, even for this simple model ( Table 1). libBi uses a DSL to define both the update and observation functions, which are transpiled into C++, then compiled into a standalone executable supporting all its inference methods. We implemented an identical stochastic SIR model in all three packages. With compiled functions, all three methods run at similar speeds ( Table 1). 

**Table 1.** Comparison of SIR model implementations. CPU time is for a run of 10 ^6^ steps with *n _P_* = 10; *S*_0_ = 10 ^6^; *I*_0_ = 10; *dt* = 10 ^−4^ on a single core. We focus on a long simulation time, as profiling of the more realistic COVID-19 transmission model showed most computation time during inference was spent in this stage, and minimises measurement of overheads.

Package	Potential parallelisation	CPU time	Model language	Interface language
dust	*n _P_m _c_*	0.96s	R/odin DSL	R
pomp	*m _c_*	82s	R	R
pomp	*m _c_*	1.3s	C	R
libBi	*n _P_m _c_*	1.04s	libBi DSL	bash

The main differences between pomp and our packages are:

- In pomp, parallelisation is only over independent chains *m _c_*; parallelisation of particles is unsupported.
- To write efficient code in pomp, users must write directly in C, no DSL is available. This also makes function debugging more challenging as there is no built-in parser.
- Automated generation of code for arrays is not supported in pomp.
- Parameter constraints are not directly supported in pomp and must instead be implemented through monotonic transforms, such as taking the logarithm.
- pomp has additional maximisation routines available.

The main differences between libBi and our packages are:

- The interface to libBi is through the command line, and parameters are set through configuration files. There is no direct interface to R.
- Parameter setup to be used for both simulation and inference in libBi may require different model definitions.
- Combining inference and forecasting tasks is possible, but requires chaining configuration files in libBi rather than relying on object reuse.
- In libBi, Input and output is in the NetCDF format, which is efficient and compact, but requires extra tools and knowledge to manipulate, and is not human-readable.
- All functions must be written in the libBi DSL, and although this is extensive, it is still more restrictive than a full language such as R, as may be used in pomp and our packages. However, this also allows faster compiled code to be generated for the observation functions.
- An alternative method of parallel random number generation is used in libBi ^43, 44^, and further parallelisation over parameters is additionally supported when using the SMC ^2 ^ algorithm for inference.
- libBi also has maximisation routines available.

Overall we would summarise these three packages as being broadly similar in purpose and efficiency. We believe that the major advantages of our packages are the tight interface with R, the easy-to-use DSL, and the considered parallel simulation machinery. In comparing these packages, anecdotally we found the flow of data between the source and models was simplest in our packages – for our rapidly evolving COVID-19 model, this made our packages the only viable choice. The growing set of auxiliary functions specific to COVID-19 modelling in the sircovid package are easily referenced between packages, demonstrating that being close to the language users are conversant with has a clear interface advantage. However, we expect that different users will have different preferences for these packages. Users will now have three good software choices available, which they can decide between based on their background and needs.

## Summary

State space models are broadly used to model biological processes, particularly transmission of infectious diseases. In principle a simple state space model can readily be implemented in any number of programming languages, but keeping this model efficient, reproducible and correct, especially as it is expanded to include more processes and complexity, is a cross-disciplinary challenge. We have produced a suite of packages intended to make the mechanics of model development and fitting as simple and efficient as possible, so modellers can focus on the biology of their problem, rather than spending time on software integration challenges.

Software solutions must balance the competing needs of modellers, statisticians and software developers, thus tradeoffs necessarily exist. Anecdotal experience with expert modellers led to the following design requests: use of a DSL close to R to make compiled code; reproducibility of results; parallelisation with a fair random number draws for simulation; tight integration with the R language to allow easier definition of an observation function; fast implementation of new inference methods, especially when adding compartments or age-structure; and more flexible uses of the model simulator. 

We aimed to produce methods which lie closer to the typical skill set and scientific interest of epidemiologists than previous state space modelling packages. Resulting model objects are lightweight and directly connected to R, making their reuse easy and flexible. No advanced programming skills are required to use the packages, and the definition of likelihood functions in R itself means that in practice few restrictions are placed on models, other than that they are Markovian. Optimised code for model simulation is automatically generated, and modern Bayesian methods such as SMC can be applied without needing a thorough understanding of the mechanics of their operation. Models are guaranteed to be reproducible, ‘play fair’ with randomness even when parallelised, and come with a suite of fully unit-tested inference methods. CPU parallelisation is efficient, and the code developed here will form the basis of future speedups using specialist hardware such as general purpose graphics processing units.

These packages helped us with reliability, speed of model development, and the speed of real-time inference in our model of COVID-19 transmission in the UK. Due to their generality, we believe these packages will be more broadly useful for a range of modelling attempts, and will mean modellers do not have to reinvent the wheel each time a new model and inference method is produced.

## Software availability

Software available from:

- odin: https://mrc-ide.github.io/odin
- odin.dust: https://mrc-ide.github.io/odin.dust
- dust: https://mrc-ide.github.io/dust
- mcstate: https://mrc-ide.github.io/mcstate

Source code available from:

- odin: https://github.com/mrc-ide/odin
- odin.dust: https://github.com/mrc-ide/odin.dust
- dust: https://github.com/mrc-ide/dust
- mcstate: https://github.com/mrc-ide/mcstate
- Plots: https://github.com/mrc-ide/odin-dust-plots

Archived source code as at time of publication:

- odin: https://doi.org/10.5281/zenodo.4298476 ^45^
- odin.dust: https://doi.org/10.5281/zenodo.4297456 ^46^
- dust: https://doi.org/10.5281/zenodo.4298491 ^47^
- mcstate: https://doi.org/10.5281/zenodo.4298607 ^48^
- Plots: https://doi.org/10.5281/zenodo.4293396 ^49^

Software license: MIT
