# Supplementary material for: Reproducible parallel inference and simulation of stochastic state space models using odin, dust, and mcstate
Source: Wellcome Open Res. 2021 Jun 10;5:288. Originally published 2020 Dec 11. [Version 2] doi: 10.12688/wellcomeopenres.16466.2 (PMC8552050; doi:10.12688/wellcomeopenres.16466.2)
Supplement: Peer review history

# Peer review: Reviewer response for version 2

**DOI:** 10.21956/wellcomeopenres.18728.r44387

**Published:** 2021-10-27

**Authors:** Edward Ionides

**Affiliations:** 1 Department of Statistics, University of Michigan, Ann Arbor, MI, USA

I've looked through the revision, which is thorough. Please update the status of my recommendation to "Approved". I did notice many capitalization errors in the references - perhaps an artifact of using BibTex? I recommend these are fixed, though it is not scientifically important.”

Are the conclusions about the tool and its performance adequately supported by the findings presented in the article?

Yes

Is the rationale for developing the new software tool clearly explained?

Yes

Is the description of the software tool technically sound?

Yes

Are sufficient details of the code, methods and analysis (if applicable) provided to allow replication of the software development and its use by others?

Yes

Is sufficient information provided to allow interpretation of the expected output datasets and any results generated using the tool?

Yes

Reviewer Expertise:

NA

I confirm that I have read this submission and believe that I have an appropriate level of expertise to confirm that it is of an acceptable scientific standard.

# Peer review: Reviewer response for version 1

**DOI:** 10.21956/wellcomeopenres.18131.r41899

**Published:** 2021-01-26

**Authors:** Rene Niehus

**Affiliations:** 1 Center for Communicable Disease Dynamics, Department of Epidemiology, Harvard T H Chan School of Public Health, Boston, MA, USA

Knock *et al*. have developed a software that allows building and fitting of state space models to (partially) observed data and that allows simulation from those models. Like tools such as WinBugs or Stan their tool allows the user to define models in language that is similar to statistical modelling language. It is efficient in fitting time-series via transcribing the model into fast C++ language, parallelisation, and the use of particle filtering, similar to pomp or libBo. It integrates well with commonly used R environment, and it is thus available to use for a wide group of researchers.

 I would not be surprised if this software and its iterations took an important role for predicting and understanding the ongoing COVID-19 pandemic. A key determinant for this is its development and refinement by an interdisciplinary team, and working with feedback from use case in an urgent public health crisis.

 I truly enjoyed reading this paper, and I would like to suggest a few points of improvement, before I make some suggestions for improved readability.

1. The authors describe at first some general concepts, then the different components of their framework, the random number generator, its operation, and finally a comparison with other tools. It should be made clearer at the very beginning, in what ways this software advances current tools, and combines the advantages of different tools. It remains unclear if the dust-object idea containing particles is novel, or an adaptation. I also suggest extending Table 1 to include several of the other features that differ between dust, pomp, and libBi.
2. The tool of the authors follows a Bayesian framework, which I consider especially suitable in the context of COVID-19 modelling where information on biological parameters is added constantly through various trials. I am not entirely sure, but I think the Figure 3 shows a prior-predictive simulations/checks: Once a user has written the structure of a model and they want to understand the implications of the prior choice on the outcome-scale, then they would want to simulate from the model and its priors (instead of posteriors)(see McElreath 2020, Chapter 4.3.2). As this is one of the strengths of this simulation-based tool, it would be useful if the authors added prior predictive checks as an explicit step in the described work flow. 
3. Following the above point, I am wondering if the user is limited to priors in the shape of standard distributions (e.g., Normal(0,1) ), or would it be possible for the user to define a prior based on a posterior from a different model, for example in the form of 1000 random draws of the posterior? While I see how this is a computational challenge, I think it is a feature that might be very useful for adding results from other Bayesian studies. Can the authors at least comment on this?
4. Is it possible to include time varying variables, such as a R0 that is changing in time? It is not clear from the text if this is possible. If yes, a reader might benefit from advise on how to smooth such a variable over time to avoid overfitting.
5. It would be very helpful to have a cartoon that visualises how odi/dust/mcstate work together and how they make use of the abstractions (particle and dust) and how all this system generates the model building pipeline. 

As this paper is meant to help researchers like for example infectious disease modellers to make use of sophisticated software, it will be useful to revise the text to help this target audience to better follow the paper. Here are some concrete ideas:

- A non-technical reader will not gain much from Figure 1 without further comparisons or explanation. What does a straight line signify? Should this surprise, what is to expect?
- Briefly explain what a dynamics library is.
- Briefly explain why C++ is used at the backend (speed).
- Briefly explain (or avoid) the term transpile.
- “change in randomness” page 6 should be explained more precisely.
- Redefine what “m” mean on page 6.
- The indexing in the expression m_{i} seems odd if m is defined as in integer above. Please explain or change.
- Is the variance-covariance matrix for the proposal kernel computed in an automated step, or does the user have to do this, or make that setting?
- Adding example code that uses the predict() function would help.
- “adding square brackets to the left hand side of each declaration”, a reference to a code block should be added. It would help to enumerate the code blocks for easy reference.
- It is not clear how entries of the contact matrix m are defined 
- Equation (1) needs definition of beta, N, I and m.

I found a few typos

 “psuedorandom numbers” page 4

 “first dimension[s]” page 12

 “and and” page 15

Are the conclusions about the tool and its performance adequately supported by the findings presented in the article?

Yes

Is the rationale for developing the new software tool clearly explained?

Partly

Is the description of the software tool technically sound?

Yes

Are sufficient details of the code, methods and analysis (if applicable) provided to allow replication of the software development and its use by others?

Yes

Is sufficient information provided to allow interpretation of the expected output datasets and any results generated using the tool?

Yes

Reviewer Expertise:

Infectious disease epidemiology, COVID-19 dynamics, microbiome dynamics.

I confirm that I have read this submission and believe that I have an appropriate level of expertise to confirm that it is of an acceptable scientific standard.

## References

[1] : Statistical rethinking: A Bayesian course with examples in R and Stan. *CRC press* .Mar 2020;

# Response

**Published:** 2021-06-03

**Authors:** John Lees

**Affiliations:** Imperial College London, UK

*Knock et al. have developed a software that allows building and fitting of state space models to (partially) observed data and that allows simulation from those models. Like tools such as WinBugs or Stan their tool allows the user to define models in language that is similar to statistical modelling language. It is efficient in fitting time-series via transcribing the model into fast C++ language, parallelisation, and the use of particle filtering, similar to pomp or libBo. It integrates well with commonly used R environment, and it is thus available to use for a wide group of researchers.*

*I would not be surprised if this software and its iterations took an important role for predicting and understanding the ongoing COVID-19 pandemic. A key determinant for this is its development and refinement by an interdisciplinary team, and working with feedback from use case in an urgent public health crisis.*

*I truly enjoyed reading this paper, and I would like to suggest a few points of improvement, before I make some suggestions for improved readability.*

 We thank the reviewer for their kind comments and positive summary. We have a number of additional changes to the text which we believe should clarify the points arising.

*The authors describe at first some general concepts, then the different components of their framework, the random number generator, its operation, and finally a comparison with other tools. It should be made clearer at the very beginning, in what ways this software advances current tools, and combines the advantages of different tools. It remains unclear if the dust-object idea containing particles is novel, or an adaptation. I also suggest extending Table 1 to include several of the other features that differ between dust, pomp, and libBi.*

**

 We are somewhat constrained by the software manuscript format: demonstrating motivation and specific use cases before a full comparison with other methods – so we have left the full comparison with the alternative packages before the summary. However, we do agree that this would be useful to be introduced earlier on, so have added more text to the introduction to clarify the differences between packages, and expanded table 1 as suggested.

 We are not sure of the exact manner in which pomp and libBi are engineering in this regard, but this kind of class definition is a fairly typical occurrence in C++ libraries. But, we have added more of a description of why we opted for the Dust/Particle separation, which we think makes the rationale for this decision clearer, and was missing previously. We have also added and reference a “design” vignette, which describes this in further detail.

*The tool of the authors follows a Bayesian framework, which I consider especially suitable in the context of COVID-19 modelling where information on biological parameters is added constantly through various trials. I am not entirely sure, but I think the Figure 3 shows a prior-predictive simulations/checks: Once a user has written the structure of a model and they want to understand the implications of the prior choice on the outcome-scale, then they would want to simulate from the model and its priors (instead of posteriors)(see McElreath 2020, Chapter 4.3.2). As this is one of the strengths of this simulation-based tool, it would be useful if the authors added prior predictive checks as an explicit step in the described work flow.*

 We now follow the reviewer’s suggestion, and add a section on simulating from the priors to the workflow. Notably, we have added a simulate method to the dust package which makes running models across the time series using a specific set of parameters easier and more flexible.

**

*Following the above point, I am wondering if the user is limited to priors in the shape of standard distributions (e.g., Normal(0,1) ), or would it be possible for the user to define a prior based on a posterior from a different model, for example in the form of 1000 random draws of the posterior? While I see how this is a computational challenge, I think it is a feature that might be very useful for adding results from other Bayesian studies. Can the authors at least comment on this?*

**

 One advantage of our framework’s tight integration with R is that functions for the likelihood and prior are completely flexible, and can use any functionality the R language allows. Although we demonstrate with a simple prior distribution in the use cases, any function can be defined. This can include using functions from external packages, or, as suggested, drawing from another model’s posterior. We’ve noted this more specifically in the revised text. A future feature will include the ability to ‘restart’ models from part way along the time series, and will draw on this suggestion to re-initialise the model.

*Is it possible to include time varying variables, such as a R0 that is changing in time? It is not clear from the text if this is possible. If yes, a reader might benefit from advise on how to smooth such a variable over time to avoid overfitting.*

**

 Indeed, this is possible, and used extensively in the SIRCOVID package. To do so in these packages usually requires adding a simple ‘transform’ function in the particle filter, which translates between the parameters being inferred in R, and parameters in the model code. The easiest way to implement time-varying parameters is to make them piecewise-linear, and point the reader to the example in SIRCOVID of how to set this up (but do add a paragraph noting this to the examples). In theory a smoothing method such a spline fitting using a few free parameters would also be possible, but we do not demonstrate that here.

*It would be very helpful to have a cartoon that visualises how odin/dust/mcstate work together and how they make use of the abstractions (particle and dust) and how all this system generates the model building pipeline.*

 We have added an extra figure giving an overview of the software packages.

*As this paper is meant to help researchers like for example infectious disease modellers to make use of sophisticated software, it will be useful to revise the text to help this target audience to better follow the paper. Here are some concrete ideas:*

 Thank you for these suggestions, which we address individually below. We expect that many modellers will find the package vignettes particularly useful in addition to this paper – there we are able to separate out technical language on design decisions with practical guides on how to use the software.

*A non-technical reader will not gain much from Figure 1 without further comparisons or explanation. What does a straight line signify? Should this surprise, what is to expect?*

**

 Added to caption.

*Briefly explain what a dynamics library is.*

 Added.

**

*Briefly explain why C++ is used at the backend (speed).*

 Added.

**

*Briefly explain (or avoid) the term transpile.*

 Added.

**

*“change in randomness” page 6 should be explained more precisely.*

 Explanation added.

**

*Redefine what “m” mean on page 6.*

*The indexing in the expression m_{i} seems odd if m is defined as in integer above. Please explain or change.*

 Thanks for spotting this – this should have been *p*, indexed for each core/thread not chain.

**

*Is the variance-covariance matrix for the proposal kernel computed in an automated step, or does the user have to do this, or make that setting?*

 This is indeed a user option. We note that in the text, and a package vignette discusses how to set this in more detail than we have space for in this paper.

**

*Adding example code that uses the predict() function would help.*

 Added.

**

*“adding square brackets to the left hand side of each declaration”, a reference to a code block should be added. It would help to enumerate the code blocks for easy reference.*

 Reference to code block added. We are unfortunately unable to give line numbers to the code blocks due to formatting constraints.

**

*It is not clear how entries of the contact matrix m are defined*

**

 We have reordered the text slightly, and expanded the description.

*Equation (1) needs definition of beta, N, I and m.*

*I found a few typos*

*“psuedorandom numbers” page 4*

*“first dimension[s]” page 12*

*“and and” page 15*

 Typos fixed.

# Peer review: Reviewer response for version 1

**DOI:** 10.21956/wellcomeopenres.18131.r42058

**Published:** 2021-01-20

**Authors:** Edward Ionides

**Affiliations:** 1 Department of Statistics, University of Michigan, Ann Arbor, MI, USA

The authors have built a framework for model development and statistical inference for complex partially observed stochastic dynamic models, motivated by applications in epidemiology. The methods are simulation-based, offering scientists appealing flexibility to investigate a range of different model specifications. The resulting odin/dust/mcstate software environment is compared with two other packages having similar capabilities, libBi and pomp. Time comparisons are comparable, but odin/dust/mcstate has had considerable work put into some important practical considerations: (i) the domain-specific language (DSL) used for model specification; (ii) parallelization issues.

 The work undertaken by the authors contributes a new perspective to the worthwhile task of building and testing models to interpret data on noisy and incompletely measured dynamic systems. I shall compare odin/dust/mcstate to my experiences with pomp (King *et al*, 2016) ^1^ raising some points that are missing from the current version of the authors' manuscript.

1. The authors emphasize only Bayesian inference. The Bayesian paradigm is convenient for combining uncertainty about parameters with variability inherent in the dynamics, making it well placed to provide forecasts once the prior and model are satisfactory. However, in some situations there are advantages to avoiding the additional requirement to add to the model a quantification of prior uncertainty about parameters. For model criticism, the goal is to diagnose features of the model that are incompatible with the data. If such features exist, then a compatible prior does not exist and so the requirement to specify one is a hindrance rather than a help. It may be better to first investigate model specification by cycles of the scientific method promoted by Popper (1959) ^2^: What features of the data are inconsistent with the hypothesized model? Can we find a better model?
2. Maximum likelihood estimation, and associated likelihood ratio tests and profile likelihood confidence intervals, provide non-Bayesian methodology for partially observed stochastic dynamic models that also make statistically efficient use of data. The odin/dust/mcstate framework could, for example, implement an iterated filtering likelihood maximization (Ionides *et al*, 2015) ^3^ as has been much used in pomp.
3. Monte Carlo adjusted profile likelihood estimation (Ionides *et al*, 2017) ^4^ has some favorable scaling properties (Ning *et al*, 2020) ^5^ that are not shared by particle Markov chain Monte Carlo (Doucet *et al*, 2015) ^6^. For analysis that stretches available computational resources, computational scaling may be another reason to consider likelihood-based inference.
4. I agree with the authors on the importance of developing software accessible to a broad technical audience. The DSL written by the authors builds on the success of packages such as WinBUGS, JAGS, and stan. The Csnippet approach used by pomp is somewhat different: it gives the users the full flexibility of C while hiding certain details such as variable definitions for latent states, observations and parameters. For simple models, pomp Csnippet code can look very much like WinBUGS DSL code. For more complex models, the user may start needing to use increasingly esoteric properties of C, but this is a strength as well as a weakness, since it is hard to anticipate and encode in a DSL all the features one might want in a simulation model. The Csnippet framework is simple enough that it is accessible to students in a Masters level time series course where students with no prior C experience develop and fit their own pomp analysis.
5. The benefits of parallelizing the particle filter depend on the purpose to which it is put. If it is used in a single, long, particle Monte Carlo Markov chain calculation then parallelization may help. However, in practice, one should carry out many replications of the inferential algorithm while varying the starting point and the seed of the random number generator. In a likelihood-based framework, one may want to evaluate and maximize the likelihood at a range of profile points. In these cases, parallelizing within the particle filter can be inferior to parallelizing over the embarassingly parallel replicated filters.

Are the conclusions about the tool and its performance adequately supported by the findings presented in the article?

Yes

Is the rationale for developing the new software tool clearly explained?

Yes

Is the description of the software tool technically sound?

Yes

Are sufficient details of the code, methods and analysis (if applicable) provided to allow replication of the software development and its use by others?

Yes

Is sufficient information provided to allow interpretation of the expected output datasets and any results generated using the tool?

Yes

Reviewer Expertise:

Time series analysis, with applications in epidemiology and ecology.

I confirm that I have read this submission and believe that I have an appropriate level of expertise to confirm that it is of an acceptable scientific standard, however I have significant reservations, as outlined above.

## References

[1] : Statistical Inference for Partially Observed Markov Processes via theR Packagepomp. *Journal of Statistical Software* .2016;69(12) : 10.18637/jss.v069.i12 10.18637/jss.v069.i12

[2] : The Logic of Scientific Discovery. *Hutchinson* .1959;

[3] : Inference for dynamic and latent variable models via iterated, perturbed Bayes maps. *Proc Natl Acad Sci U S A* .2015;112(3) : 10.1073/pnas.1410597112 719-24 10.1073/pnas.1410597112 25568084PMC4311819

[4] : Monte Carlo profile confidence intervals for dynamic systems. *J R Soc Interface* .14(132) : 10.1098/rsif.2017.0126 10.1098/rsif.2017.0126 28679663PMC5550967

[5] : Scalable Monte Carlo Inference and Rescaled Local Asymptotic Normality. *arXiv* .2020; Reference source

[6] : Efficient implementation of Markov chain Monte Carlo when using an unbiased likelihood estimator. *Biometrika* .2015;102(2) : 10.1093/biomet/asu075 295-313 10.1093/biomet/asu075

# Response

**Published:** 2021-06-03

**Authors:** John Lees

**Affiliations:** Imperial College London, UK

*The authors have built a framework for model development and statistical inference for complex partially observed stochastic dynamic models, motivated by applications in epidemiology. The methods are simulation-based, offering scientists appealing flexibility to investigate a range of different model specifications. The resulting odin/dust/mcstate software environment is compared with two other packages having similar capabilities, libBi and pomp. Time comparisons are comparable, but odin/dust/mcstate has had considerable work put into some important practical considerations: (i) the domain-specific language (DSL) used for model specification; (ii) parallelization issues.*

*The work undertaken by the authors contributes a new perspective to the worthwhile task of building and testing models to interpret data on noisy and incompletely measured dynamic systems. I shall compare odin/dust/mcstate to my experiences with pomp (King et al, 2016) ^1^ raising some points that are missing from the current version of the authors' manuscript.*

 We thank the reviewer for their comments and thoughtful suggestions. We have made two major additions to the code to add the functionality as suggested, and modified the text to better address the issues raised. We have also added some of this summary into our introduction, to introduce pomp and libBi earlier on.

*The authors emphasize only Bayesian inference. The Bayesian paradigm is convenient for combining uncertainty about parameters with variability inherent in the dynamics, making it well placed to provide forecasts once the prior and model are satisfactory. However, in some situations there are advantages to avoiding the additional requirement to add to the model a quantification of prior uncertainty about parameters. For model criticism, the goal is to diagnose features of the model that are incompatible with the data. If such features exist, then a compatible prior does not exist and so the requirement to specify one is a hindrance rather than a help. It may be better to first investigate model specification by cycles of the scientific method promoted by Popper (1959) ^2^: What features of the data are inconsistent with the hypothesized model? Can we find a better model?*

 It is true that we focused on Bayesian inference using a prior and model which have been determined to be satisfactory, which leaves out the model criticism phase. We have now added two sections on model criticism, alternatives to prior specification, and maximum likelihood estimation (noting the additions in response to the second point). We hope this expands to scope of discussion here suitably.

**

*Maximum likelihood estimation, and associated likelihood ratio tests and profile likelihood confidence intervals, provide non-Bayesian methodology for partially observed stochastic dynamic models that also make statistically efficient use of data. The odin/dust/mcstate framework could, for example, implement an iterated filtering likelihood maximization (Ionides et al, 2015) ^3^ as has been much used in pomp.*

 Thank you for this suggestion, we agree that this would be a nice addition, and fits well with the design of our packages. We have now implemented and tested this algorithm using dust and mcstate. We have also added a package vignette and section to this paper describing its use.

*Monte Carlo adjusted profile likelihood estimation (Ionides et al, 2017) ^4^ has some favorable scaling properties (Ning et al, 2020) ^5^ that are not shared by particle Markov chain Monte Carlo (Doucet et al, 2015) ^6^. For analysis that stretches available computational resources, computational scaling may be another reason to consider likelihood-based inference.*

 We hope that our additions to the text and the addition of the iterated filtering algorithm now allow likelihood-based inference in our packages. Although we have not added the MCAP algorithm to the package code, we have added this point and associated references to the text, as is done in pomp.

*I agree with the authors on the importance of developing software accessible to a broad technical audience. The DSL written by the authors builds on the success of packages such as WinBUGS, JAGS, and stan. The Csnippet approach used by pomp is somewhat different: it gives the users the full flexibility of C while hiding certain details such as variable definitions for latent states, observations and parameters. For simple models, pomp Csnippet code can look very much like WinBUGS DSL code. For more complex models, the user may start needing to use increasingly esoteric properties of C, but this is a strength as well as a weakness, since it is hard to anticipate and encode in a DSL all the features one might want in a simulation model. The Csnippet framework is simple enough that it is accessible to students in a Masters level time series course where students with no prior C experience develop and fit their own pomp analysis.*

 Thank you for this perspective on the Csnippet approach. We have expanded our discussion of the DSL to note the strengths and weaknesses of either approach from a user point of view.

 If greater flexibility is required, we also note that it is possible to directly write C/C++ code to be used as a dust model (and therefore with the rest of our framework). The easiest way of doing this is by modifying one of our examples – by just changing single sections of the model definition this ends up being somewhat similar to the Csnippet approach. For testing some models and new features of dust, this is the route we took. Our experience with real-life complex models has been that the odin DSL was greatly preferred due to its built-in bookkeeping of model indices, and did not limit any of the required features.

 As a further alternative, we have also added the ability to the odin DSL to call arbitrary C++ functions – essentially imitating the Csnippet approach where the DSL may prove limiting.

*The benefits of parallelizing the particle filter depend on the purpose to which it is put. If it is used in a single, long, particle Monte Carlo Markov chain calculation then parallelization may help. However, in practice, one should carry out many replications of the inferential algorithm while varying the starting point and the seed of the random number generator. In a likelihood-based framework, one may want to evaluate and maximize the likelihood at a range of profile points. In these cases, parallelizing within the particle filter can be inferior to parallelizing over the embarassingly parallel replicated filters.*

 This is an excellent point, and as such we have now expanded the flexibility of the parallelisation. Users can now parallelise both over particles and over filters independently, and choose how to do so depending on their problem. The interface is straightforward – between chain parallelism is enabled by increasing the number of workers, and specifying a total number of threads available.

 The issue of varying the starting point is addressed by the ‘initial’ argument to the particle filter, through which the user can specify an arbitrary R function to generate the initial conditions for the filter.

 We have added the pmcmc_chains_prepare() function to address the issue of varying the starting seed. This uses the ‘long jump’ feature of the Xoshiro random number generator to generate streams which are uncorrelated even when further parallelisation is used within them. This function automates both the process of initialising a set of chains with different (and uncorrelated) seeds and initial conditions.

 We concur that parallelising within the particle filter can be inferior to parallelising chains – in some cases we have observed up to a 40% loss of efficiency (mostly due to particle divergence). We have found the added flexibility on this matter of great practical use in large problems on large distributed systems. Using code to orchestrate and collect information from independent runs, we can now run independent particle filters on separate computer nodes, and nested within these parallelise further over particles on each shared memory node. This top level of parallelisation can also be used to run with different parameters, or using the same model with different data (for example, fitting a COVID-19 model to different regions).
